# Amyloid-Associated Nucleic Acid Hybridisation

**DOI:** 10.1371/journal.pone.0019125

**Published:** 2011-05-19

**Authors:** Sebastian Braun, Christine Humphreys, Elizabeth Fraser, Andrea Brancale, Matthias Bochtler, Trevor C. Dale

**Affiliations:** 1 School of Biosciences, Cardiff University, Cardiff, Wales, United Kingdom; 2 School of Pharmacy, Redwood Building, Cardiff University, Cardiff, Wales, United Kingdom; 3 School of Chemistry, Cardiff University, Cardiff, Wales, United Kingdom; 4 International Institute of Molecular and Cell Biology (IIMCB), Warsaw, Poland; New England Biolabs, Inc., United States of America

## Abstract

Nucleic acids promote amyloid formation in diseases including Alzheimer's and Creutzfeldt-Jakob disease. However, it remains unclear whether the close interactions between amyloid and nucleic acid allow nucleic acid secondary structure to play a role in modulating amyloid structure and function. Here we have used a simplified system of short basic peptides with alternating hydrophobic and hydrophilic amino acid residues to study nucleic acid - amyloid interactions. Employing biophysical techniques including X-ray fibre diffraction, circular dichroism spectroscopy and electron microscopy we show that the polymerized charges of nucleic acids concentrate and enhance the formation of amyloid from short basic peptides, many of which would not otherwise form fibres. In turn, the amyloid component binds nucleic acids and promotes their hybridisation at concentrations below their solution *K*_d_, as shown by time-resolved FRET studies. The self-reinforcing interactions between peptides and nucleic acids lead to the formation of amyloid nucleic acid (ANA) fibres whose properties are distinct from their component polymers. In addition to their importance in disease and potential in engineering, ANA fibres formed from prebiotically-produced peptides and nucleic acids may have played a role in early evolution, constituting the first entities subject to Darwinian evolution.

## Introduction

More than 30 polypeptides including prion, Aβ and α-synuclein proteins form amyloid fibrils as an alternative ordered conformation and are thought to propagate *in vivo* by templating the misfolding of their own monomeric precursors in diseases including Creutzfeldt-Jakob, Alzheimer's and Parkinson's [1], [2]. The amyloid conformation is not limited to pathologies, but appears to be a general property of polypeptide chains [3], [4], [5]. Despite the high variation of amyloidogenic polypeptide sequences all amyloid fibres share a common core structure: a cross-β spine that comprises inter-molecular β-sheet strands running perpendicular to the fibril axis [6], [7]. Formation of amyloid is primarily mediated by main chain interactions where the probability of inter-molecular aggregation is strongly regulated by amino acid side chain identity and the environment [3]. A simple binary pattern of alternating hydrophobic and hydrophilic amino acid residues correlates with an increased propensity for amyloid formation [8], which is used to design *de novo* amyloidogenic peptides and proteins [9], [10], [11].

Pathological amyloid deposits often contain polyionic interaction partners like glycosaminoglycans, collagen and nucleic acids [12], [13], [14], [15], [16], [17]. Recent studies have shown a critical role for polyanions such as poly(A) RNA in the conversion of bacterially-expressed prion protein into infectious particles [18], [19]. The polymerized charges of nucleic acids associate with basic residues on polypeptides to concentrate and enhance their rate of amyloid formation [17]. However, the interactions of nucleic acids with amyloidogenic polypeptides are highly complex. Amyloid fibres from long peptide chains comprise discrete sequences forming the cross-β spine and unincorporated sequences that decorate the core [20], [21]. More generally, it is not known whether polyanion promotion of amyloid formation is based on direct interactions with the core cross-β spine or indirectly via the decorating sequences.

Here we use a simplified system of short basic peptides with alternating hydrophobic and hydrophilic amino acid residues to study nucleic acid - amyloid interactions. We show that nucleic acids promote amyloid formation from peptides, many of which would not otherwise form fibres. In turn, the amyloid concentrates and enhances the hybridization of associated nucleic acids. This supports the use of nucleic acid aptamers for the modulation of amyloid fibre growth in therapy and engineering. In our studies, strong reciprocal peptide-nucleic acid interactions lead to formation of amyloid-nucleic acid (ANA) complexes with discrete properties from those of their composite polymers. The formation of fibres from components present in the prebiotic environment supports a hypothesis suggesting a potential role for ANA complexes at an early stage in evolution.

## Results and Discussion

### Nucleic acids promote amyloid formation from short basic peptides

**Figure 1:**
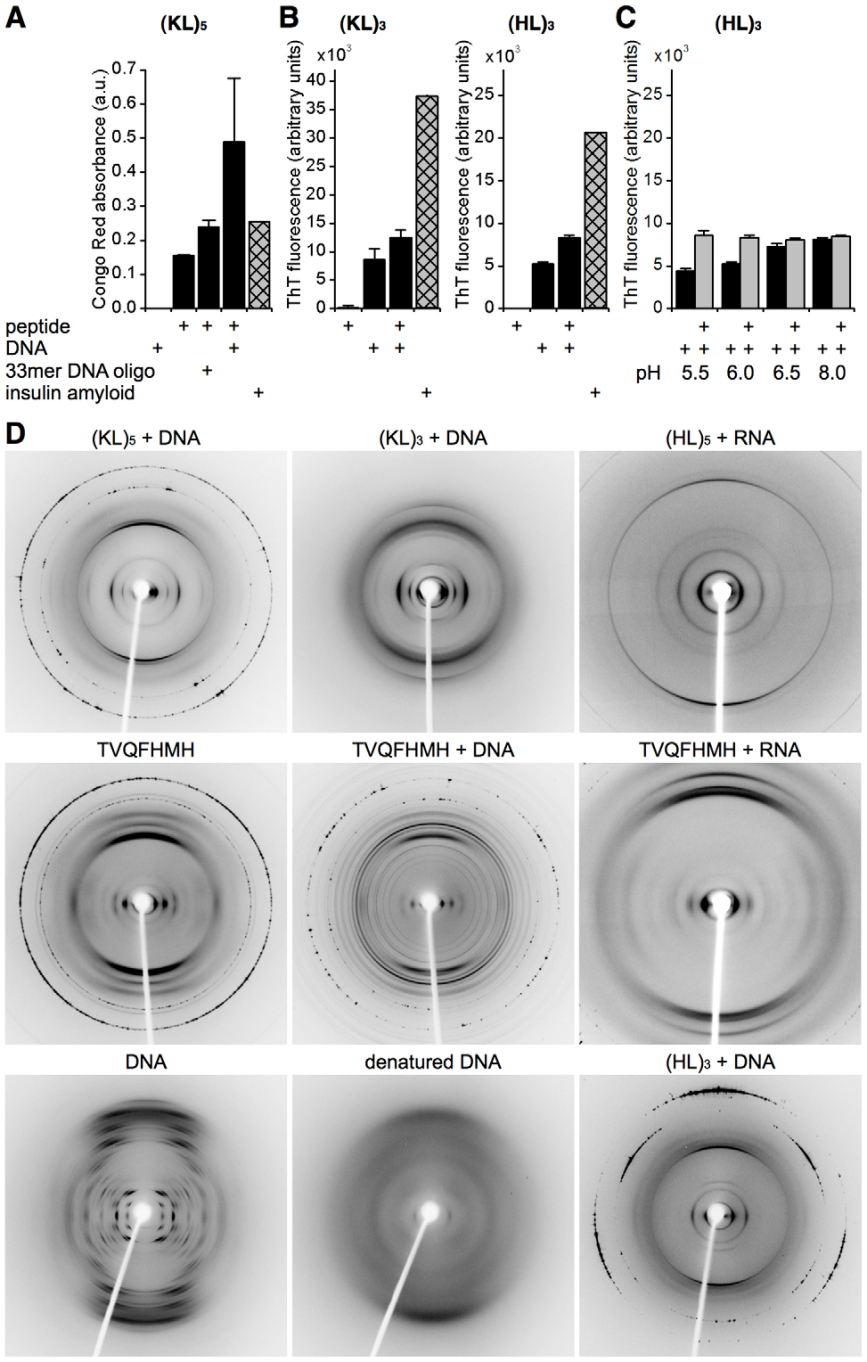
Nucleic acids promote amyloid formation by short basic peptides. (**A** to **C**) *Amyloid formation in solution*. (**A**) Congo Red absorbance at 544 nm by (KL)_5_ complexes with salmon testis or oligonucleotide DNA. (**B** and **C**) ThT fluorescence (in arbitrary units) from 17 µM (KL)_3_ or 50 µM (HL)_3_ with 50 µM DNA at varying pH. Peptide-containing samples were run in quadruplicate (mean ± s.d.), the insulin amyloid positive control was in duplicate (mean). (**D**) *X-ray fibre diffraction*. (KL)_5_, (KL)_3_, (HL)_5_, (HL)_3_, and TVQFHMH with DNA or RNA show clear cross-β patterns indicating amyloid formation. TVQFHMH was the only peptide that could be induced to show a cross-β pattern in absence of nucleic acid. Pure DNA gave a clear A-DNA diffraction pattern. Residual reflections of this pattern were not apparent within ANA complex diffraction patterns that incorporated dsDNA. The diffraction pattern of heat-denatured DNA has lost all distinctive features of the A-DNA pattern. Patterns were recorded at 180 mm detector distance except for (HL)_5_+RNA and TVQFHMH+RNA which was at 300 mm.

To better understand the relationship between nucleic acids and amyloid, we have focused on the formation of amyloid from short peptides. Peptides with alternating hydrophobic-hydrophilic residues were chosen since their presence in proteins increases the probability that they will be incorporated into amyloid [9]. (KL)_3_ and the longer (KL)_5_ were chosen as they formed gels - a characteristic of amyloid - when incubated with poly(A) RNA, but couldn't gel when incubated with equivalent levels of inorganic phosphate [22]. The related (HL)_3_ and (HL)_5_ sequences were chosen to allow modulation of peptide charge over a range of pHs that were compatible with nucleic acid hybridization. The heptamer sequence TVQFHMH was based on a sequence present within a randomly-generated amyloidogenic protein that contained tandem alternating hydrophobic/hydrophilic sequences [9]. The addition of salmon testis DNA (ST DNA) or a short 33mer oligodeoxyribonucleotide to the peptides induced an increase in Congo Red absorbance and Thioflavin T (ThT) fluorescence that has been shown to be characteristic of the formation of amyloid (Fig. 1 A–C, Fig. S1; [23]). The nucleic acid-induced changes in fluorescence correlated with rapid gel formation (Table S1, Table S2, Table S3). Charge interactions were likely to be key mediators of the nucleic acid-peptide interactions since the strength of the gels was altered by changes in ion concentration (NaCl). Similar conclusions as to the importance of charge interactions were drawn from studies of amyloidogenic proteins and polyanions [17]. Histidine-containing peptide (TVQFHMH and (HL)_3_): nucleic acid interactions were influenced by varying the pH (Table S1 and Table S2), showing greater gel formation at pH 6.2 and 6.5 than pH 5.0 where maximal peptide charge would be expected. (HL)_3_ with the highest net charge (+3 at pH 5.5; Fig. 1C) showed ThT fluorescence compared to the corresponding DNA-only control, although the intrinsic pH-dependence of nucleic acid-induced ThT fluorescence made pH titrations difficult to interpret (Fig. 1C). Previous studies have shown that short peptides only form amyloid when they have one net charge. Taken together, the observations here suggest nucleic acids enhance the propensity of singly-charged peptides to form amyloid and also promote amyloid formation from multiply-charged peptides that would not otherwise interact [11].

Using X-ray fibre diffraction we tested whether the aggregates formed the characteristic cross-β diffraction pattern that is the most direct and specific test for amyloid [24]. Strong meridional and equatorial reflections suggested the presence of amyloid with a strong reflection close to 4.75 Å in all samples that corresponded to the separation of the two hydrogen-bonded chains (Fig. 1D; Table S4). Equatorial reflections close to 10 Å were consistent with the formation of inter-sheet interactions where the specific reflections would be dependent on the van der Waals volumes of the amino acid side chains [3]. To try to identify the source of the additional reflections that were detected, we compared the diffraction patterns of the amyloid-nucleic acid (ANA) complexes with pure DNA and peptide samples prepared under similar conditions. Unfortunately, with the exception of TVQFHMH, none of the peptides could be induced to form fibres in the absence of nucleic acids (between pH 5 and 8). Incubating TVQFHMH at pH 11 followed by titration to pH 6 induced the formation of a gel that was preceded by the induction of small peptide aggregates. The nucleic acid-free TVQFHMH diffraction pattern showed a characteristic cross-β pattern that lacked some of the additional reflections found in the equivalent ANA complex. However, comparison of these additional reflections with the DNA fibre diffraction pattern did not identify patterns that were consistent with a simple co-linear alignment of amyloid and DNA fibres (Fig. 1D; Table S4).

**Figure 2:**
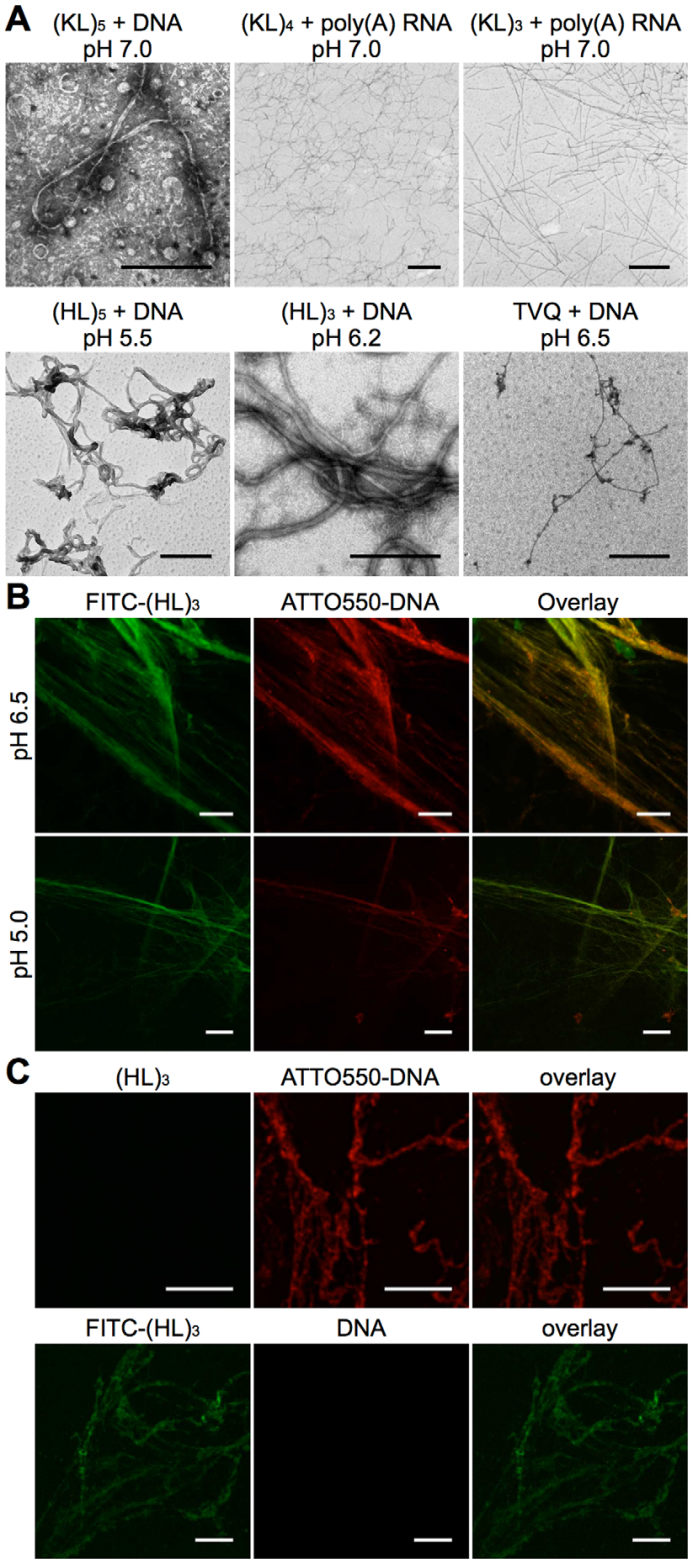
Fibre formation and colocalisation of nucleic acid and peptides in ANA complexes. (**A**) *Electron microscopy*. TEM images of ANA complexes showing the fibres formed by (KL)_5_, (HL)_5_ and TVQFHMH with DNA and (KL)_4_, (KL)_3_ and (HL)_3_ with poly(A) RNA at the indicated pHs. Scale bars are 300 nm. (**B**) *Confocal microscopy*. Representative confocal images of (HL)_3_-DNA ANA complexes where peptide and DNA samples had previously been spiked with fluorescently labelled variants. (HL)_3_ spiked 1∶20 with FITC-(HL)_3_; DNA spiked 1∶20 with nick-translated ATTO550-DNA. Peptide and DNA colocalised in large fibrillar complexes. The structures at pH 5.0 were generally finer than the ones at pH 6.5. Scale bars are 10 µM. (**C**) *Confocal control samples*. Top row: unlabelled (HL)_3_ mixed with labelled ATTO550-DNA-spiked salmon testis DNA. Bottom Row: FITC-(HL)_3_-spiked (HL)_3_ with unlabelled salmon testis DNA. No bleed-through was detected between the fluorescence channels. Scale bars are 10 µM.

**Figure 3:**
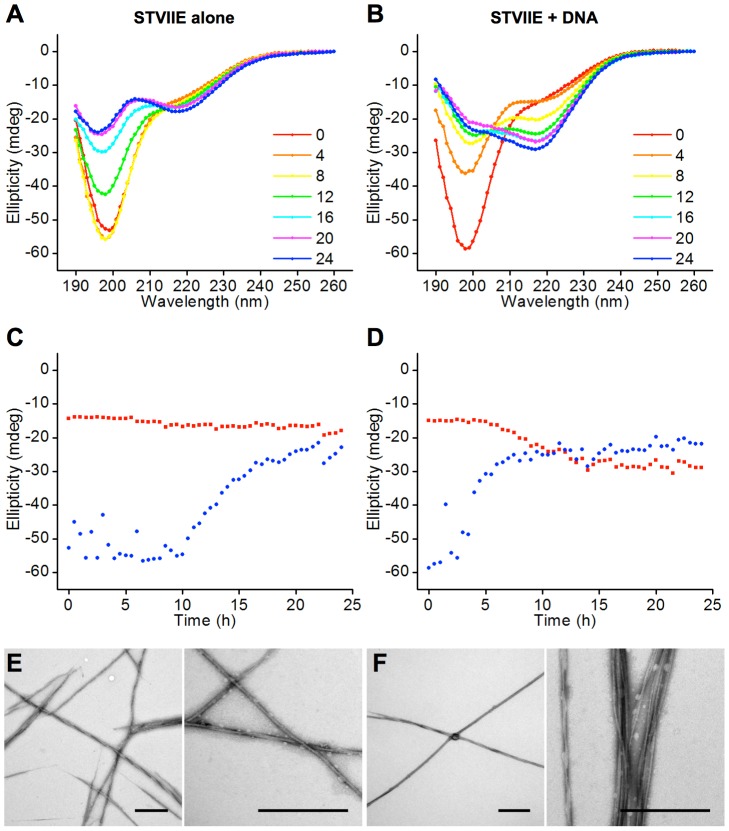
DNA oligonucleotides promote and accelerate amyloid formation. (**A** and **B**) CD spectra time course of STVIIE (0–24 h as shown) without (**A**) and with (**B**) 1∶100 DNA (charge ratio) 33mer oligonucleotide. DNA accelerates and enhances the random coil (minimum at 198 nm) to β-sheet (minimum at 218 nm) transition. (**C** and **D**) Time courses of the ellipticity at 198 nm (blue; random coil minimum) and 218 nm (red; β-sheet minimum) for STVIIE without (**C**) and with (**D**) 1∶100 DNA oligonucleotide. (**E** and **F**) Electron micrographs of STVIIE amyloid fibrils produced with (**E**) and without (**F**) 1∶100 DNA oligonucleotide. Scale bars are 500 nm.

To confirm the presence of amyloid, the ultrastructural organization of the various ANA complexes was examined by transmission electron microscopy (TEM). Fibres with minimal diameters ranging from 3.0 nm to 9.9 nm, larger diameter tapes and occasional aggregates were detected only following incubation of each of the peptides with DNA or RNA (Fig. 2A, Fig. S2, Fig. S3; Table S5). The minimal dimensions of the (KL)_5_-DNA fibres were consistent with the size of a single amyloid-nucleic acid fibre complex as modelled in Fig. S4. To determine whether the fibres were comprised of both peptide and nucleic acid, fluorescein-labelled (HL)_3_ and Atto550-labelled salmon-testis DNA were mixed with unlabelled stocks at low molar ratios (1∶20) prior to formation of the ANA complexes. All fibres detected by confocal microscopy contained co-linear peptide and nucleic acid fluorescence, suggesting intimate association between the two molecules (Fig. 2B and 2C). The absolute intensity of fluorescence in each channel varied slightly between different fibrous regions, which is probably due to incomplete mixing during the rapid formation of the gels. Taken together, the fibre diffraction, TEM and confocal microscopy data suggest that nucleic acids strongly promote the formation of amyloid from short basic peptides and the resulting ANA complexes contain nucleic acid that is closely associated with the cross-β spine of the amyloid fibril. To distinguish between ‘structural’ and ‘catalytic’ roles for nucleic acid in ANA complex formation, the effects of trace levels of nucleic acid on amyloid formation were examined by CD spectrometry. The singly charged peptide STVIIE was used because it has previously been shown to spontaneously form amyloid over a 7 day time course without the need for equimolar amounts of nucleic acid, that were found to produce a CD signal that interfered with the assessment of β-sheet formation [11]. Addition of a 1∶3300 molar ratio of a 33mer oligonucleotide to STVIIE peptide (1∶100 charge ratio) accelerated and enhanced the random to β-sheet transition while leading to formation of amyloid fibres that were indistinguishable by TEM analysis from peptide-only structures (Fig. 3). Taken together with the observation that a 1∶1 charge ratio was required for rapid gel and amyloid formation (Table S1, Table S2, Table S3, Figure S1), these data suggest that highly charged peptides require nucleic acid in both a ‘structural’ role as part of the final ANA complex, but also in a ‘catalytic’ role in the nucleation and/or extension of amyloid fibrils [17].

### Amyloid enhances nucleic acid hybridisation

**Figure 4:**
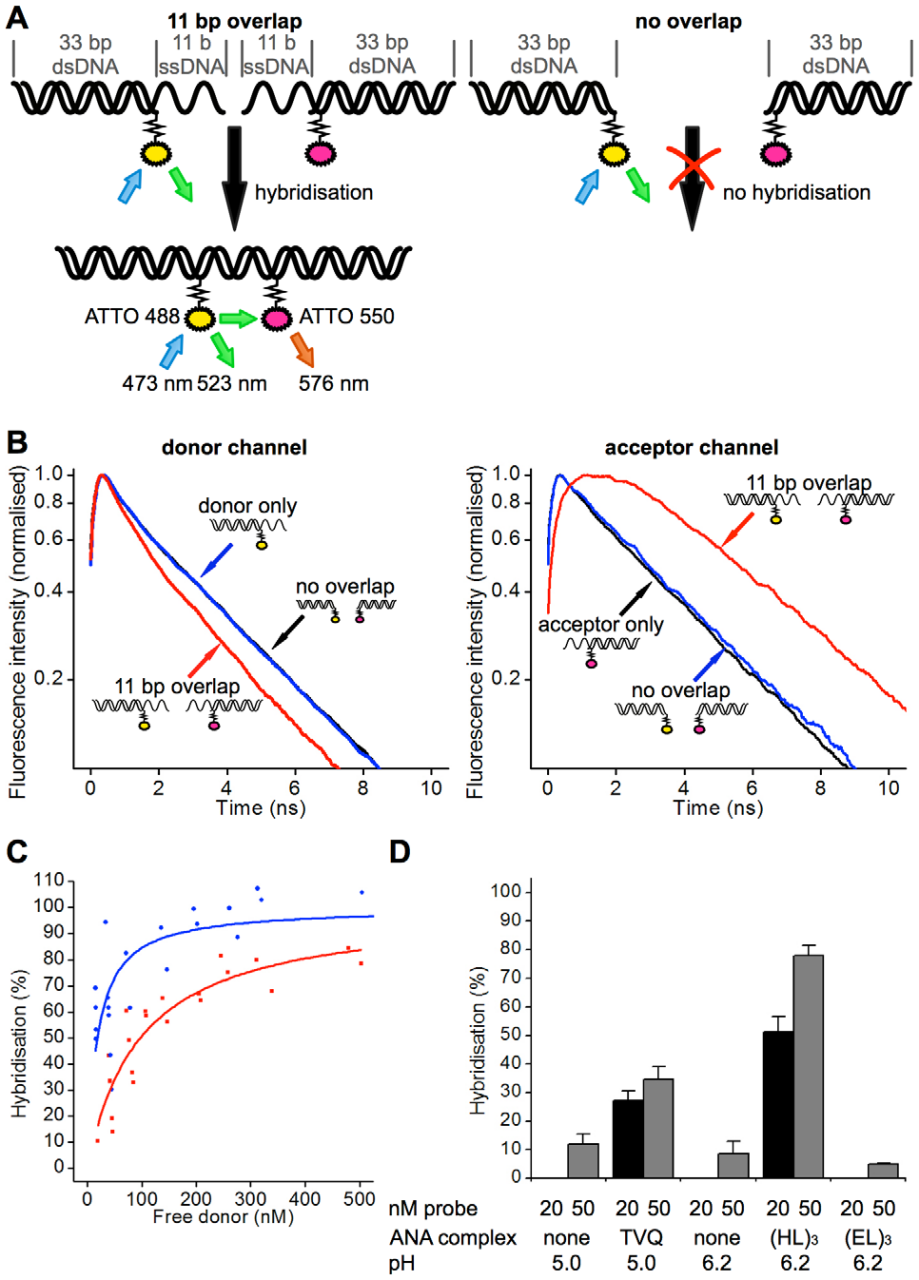
Amyloid promotes hybridisation of nucleic acids. (**A**) Schematic of the 11 bp overlap and 0 bp overlap control DNA hybridisation probes. (**B**) Faster donor and delayed acceptor fluorophore dynamics were observed with 11 bp compared to 0 bp overlap probes. Data shown from 100 nM oligonucleotides mixed with a preformed 1 mM (HL)_3_-DNA ANA complex at pH 6.2. (**C**) (HL)_3_-DNA ANA complexes enhanced the affinity of the 11 bp interaction. Pre-formed (HL)_3_ ANA complexes were mixed with probe oligonucleotides in the presence of 150 mM NaCl, 10 mM MES pH 5.5. (blue curve: (HL)_3_ ANA complexes, *K*_d_ = 18 nM±6 nM s.d. (n = 22, r = 0.48); red curve: buffer, *K*_d_ = 96 nM±23 nM s.d. (n = 21, r = 0.816)). (**D**) Basic ANA complexes increased hybridisation at low 11 bp probe oligonucleotide concentrations. (HL)_3_ and TVQFHMH, but not (EL)_3_ peptides, in the presence of DNA enhanced the hybridisation of 20 nM and 50 nM probes.

To investigate how amyloid may alter nucleic acid function, we examined whether ANA complexes could influence DNA hybridization. Results from initial approaches to measuring DNA hybridization were frustrated by the interference of the peptide components with many dsDNA detection methods. To overcome these difficulties, we used time-resolved Förster resonance energy transfer (TR-FRET) to measure the hybridization of an 11 bp ssDNA overlap between two oligonucleotide pairs (Fig. 4A). ANA complexes formed from (HL)_3_ and TVQFHMH were used because they were found not to quench the probe fluorophores and their charge could be modified by altering their pH. The histidine-containing peptides carried a +2 net charge, that in combination with a 2∶1 charge ratio (peptide: nucleic acid) generated the strongest gels (Table S1). Clear TR-FRET from the donor to the acceptor fluorophore was detected in the presence of an (HL)_3_ ANA complex showing the fluorophores were brought within the Förster radius (<6.4 nm; Figure 4B). Importantly, no TR-FRET was observed when the acceptor oligonucleotide or the single-strand DNA overlap was absent, suggesting that the TR-FRET signal represented specific hybridization (Fig. 4B). The affinity of the 11 bp interaction (in the presence of a saturating concentration of 150 mM NaCl; Fig. S5) was determined by titration of the 11 bp overlap oligonucleotides and found to be 96 nM (Fig. 4C). A similar titration showed that the presence of an (HL)_3_-DNA ANA complex strongly enhanced the affinity of the 11 bp oligonucleotides (18 nM versus 96 nM; Fig. 4C). When assayed below the *K*_d_ (20 nM or 50 nM oligonucleotides) the presence of (HL)_3_-DNA or TVQFHMH-DNA ANA complexes strongly promoted hybridization, whereas a mixture of the negatively charged peptide (EL)_3_ and DNA or additional DNA alone failed to increase hybridization (Fig. 4D). Taken together with the microscopy observation that peptides and nucleic acid are intimately associated, these data are consistent with the idea that the ANA complex rather than free peptide enhances DNA hybridization. Experimental and theoretical models have suggested that adsorption of nucleic acids to surfaces, here formed by an amyloid fibre increases the affinity of hybridization by promoting 2D diffusion [25], [26]. Furthermore, dextran-poly-L-lysine block co-polymer conjugates were suggested to promote hybridization by increasing local DNA concentrations and increasing the nucleation of hybridisation [27]. The promotion of hybridisation by charged surfaces also lies at the heart of a proposed role for clay surfaces in prebiotic evolution [28], [29].

### Was there a role for ANA fibres in prebiotic Darwinian evolution?

The data presented here support a hypothesis that suggests a role for ANA fibres in prebiotic development. In this model, growth, nucleic acid replication and division were proposed to follow simple charge and hydrophobic interactions between short prebiotically-produced nucleic acids and basic peptides [30], [31], [32], [33].

**Figure 5:**
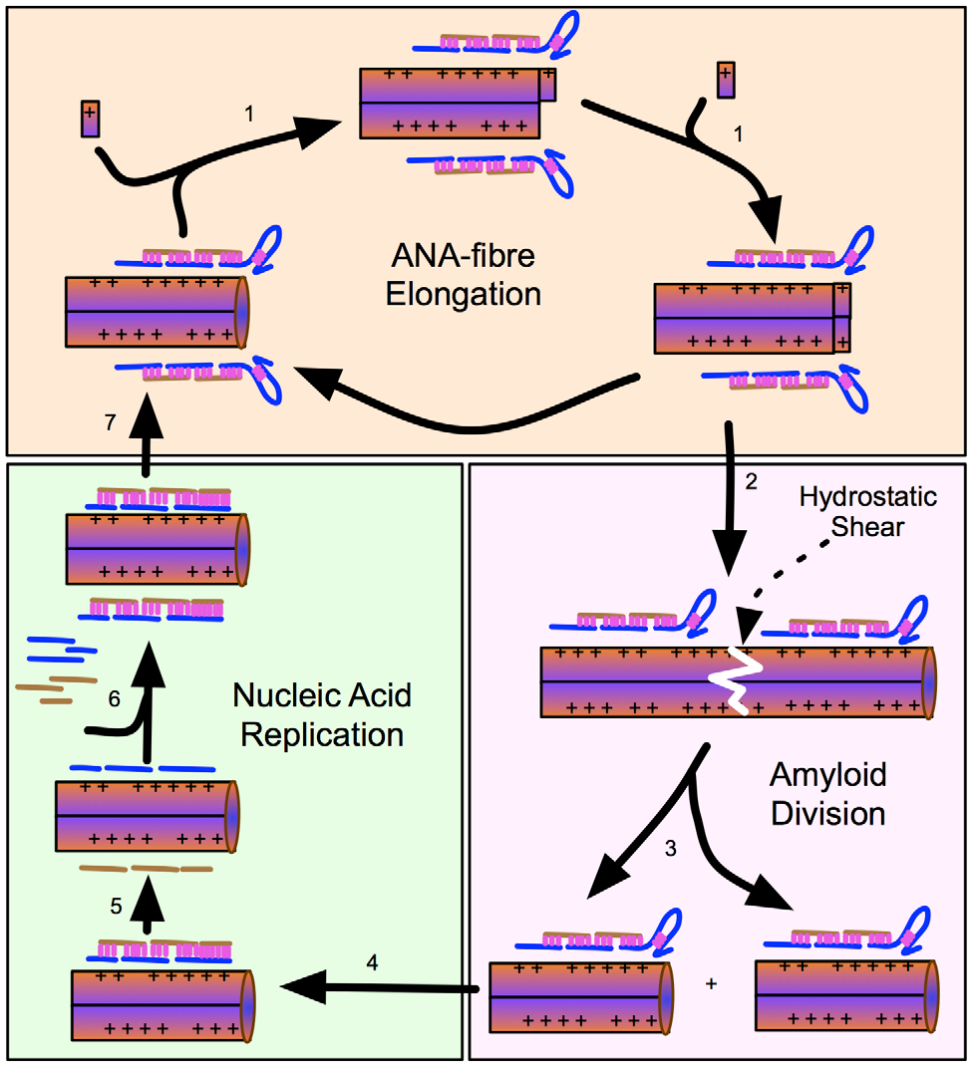
Interlocking cycles of amyloid growth and nucleic acid replication. (**1**) Nucleic acids recruit short basic amyloidogenic peptides to the growing end of the amyloid fibril primarily through charge interaction with the phosphate backbone. (**2, 3**) With increasing length, the amyloid fibrils become vulnerable to hydrostatic forces, eventually causing them to shear. This generates daughter fibrils that inherit associated and therefore related nucleic acids. (**4–6**) The amyloid fibril promotes nucleic acid replication by enhancing hybridization. (**7**) Inter and intra-molecular hybridization allows nucleic acids to adopt secondary structures, some of which may enhance the incorporation of amyloidogenic peptides. Further selection for nucleic acids that promote amyloid elongation or replication could evolve ribosome-like or polymerase/ligase-like activities respectively.

The promotion of hybridisation by charged surfaces lies at the heart of a proposed role for clay surfaces in prebiotic evolution. Clay particles have been suggested to promote hybridisation and act as compartments that enabled RNA replicases from the ‘RNA-world’ to restrict their enzymatic activity towards co-localised and therefore genetically-related nucleic acids [28], [29]. A simple extension of this model involves the idea that amyloid fibres fulfilled a similar role as a charged surface for nucleic acid replication as previously suggested [30] (Fig. 5).

An amyloid fibre that bound a nucleic acid would, unlike a clay particle, be able to grow through the recruitment of basic amyloidogenic peptides to free ends. Growing, and therefore longer, ANA fibres would be likely to be sheared by hydrostatic forces, resulting in the production of daughter fibres that would inherit related nucleic acids.

The data support aspects of this ‘ANA-world’ model of early Darwinian evolution. Fig. 1, Fig. 2, Fig. 3 show that mutual interactions between nucleic acids and peptides enhance the growth of fibres comprised of amyloid and nucleic acid (ANA fibres). Fig. 4 shows that ANA complexes can promote nucleic acid hybridisation that is a prerequisite for nucleic acid replication. Fig. S3 shows an EM image of long ANA fibres that appear to have sheared in places, consistent with the effects of hydrostatic shear promoting ANA fibre division as has previously been suggested for vesicle shearing [34].

### Conclusions

The presence of both double and single stranded nucleic acids within ANA-complexes raises the possibility that nucleic acid sequence and secondary structure could alter the rate or specificity of amyloid growth. Intriguingly, a recent study showed that *in vitro* generated PrP^sc^ amyloid selectively associated with a subset of RNA molecules to which it was exposed [18]. Multiple roles could be played by amyloid associated nucleic acids. In engineering, RNA or DNA aptamers could be used to control the growth of nanoscale fibres [20]. In therapy, similar nucleic acid structures could prevent the formation of toxic amyloid species [35]. Fibre-associated RNAzymes might be developed to block the growth of disease-associated amyloid or direct it to a less toxic species.

## Materials and Methods

### Reagents

Peptides (HL)_3_ (HLHLHL), (HL)_5_ (HLHLHLHLHL), (KL)_3_ (KLKLKL), (KL)_5_ (KLKLKLKLKL), TVQ (TVQFHMH), (EL)_3_ (ELELEL) and fluorescein-labelled FITC-(HL)_3_ were purchased at 95% purity from GL Biochem (PR China) or Peptide 2.0 (USA). Peptides were dissolved in 20 mM HCl and dried twice to remove residual TFA as previously described [36], [37] the pH was adjusted to 6.0 with NaOH and dialyzed against water (MWCO 100–500 cellulose ester membrane; Spectrum Laboratories, UK) to remove residual salt. Peptide concentrations were verified using an OPA assay for free amine groups (Pierce). Salmon testis DNA and poly(A) RNA were obtained from Sigma (UK). The peptide STVIIE was obtained HPLC-purified from Severn Biotech (UK). It was solubilized in 50% Methanol/water pH 11, freeze-dried and redissolved in 20 mM glycine pH 10 to create the stock solution. Prior to use it was sonicated for 10 minutes (bath sonicator) and centrifuged for 10 minutes at 4°C at 16,100 rcf.

### Peptide charges

The charges on the peptides were calculated using the Peptide Property Calculator provided by GenScript website (GenScript UK; www.genscript.com), resulting in the following net charges: (HL)_3_ has +1 charge at pH 6.8, +2 charges at pH 6.2, and +3 charges at pH 5.0. (HL)_5_ has ±0 charge at pH 8.0, +1 charge at pH 7.0, +2 charges at pH 6.5, +3 charges at pH 6.2, and +4 charges at pH 5.5. (KL)_3_ is at +3 charges below pH 8.5, and (KL)_5_ has +5 charges below pH 8.5. TVQFHMH has ±0 charges at pH 7.6, +1 charge at pH 6.5, +2 charges at pH 5.0. The negatively charged control peptide (EL)_3_ carries a −3 net charge in the range of the pHs explored in this paper (pH 5.0 to pH 8.0). pH titrations were carried out on several peptides to verify predictions (Fig. S6). The peptide STVIIE has previously been calculated to have a net charge of +1 at pH 2.6 [11].

### Preparation of ANA complexes

ANA complexes were formed immediately prior to assay by pipetting equal volumes of buffered peptides and nucleic acids to generate the final concentrations described. Unless otherwise indicated all DNA was double-stranded and from salmon testis and all nucleic acid concentrations relate to the molarity of the phosphates. 10 mM MES was used for assays below pH 7.0 while 10 mM HEPES was used at higher pHs. When necessary, the gels or aggregates were broken down using a pestle to allow pipetting.

### Gel strength

To evaluate gel strength, 10 µl of ANA complex solution was aspirated using a calibrated Gilson P20 pipette over a period of 2 seconds (no filter tip). A “strong gel” was defined as one that could not be pipetted since it consisted mainly of a large gel clump. A “normal strength” gel was either sucked up slower than buffer or did not reach the calibrated 10 µl level in the pipette tip. A “weak gel” was aspirated to the expected height within the 2 seconds, but showed anomalies in the meniscus indicating reduced flow.

### Dye-binding assays

Enhanced Congo Red absorbance at 544 nm of (KL)_5_ ANA complexes (0.2 mM (KL)_5_, 1 mM salmon testis or oligonucleotide DNA, 10 mM HEPES pH 8.0 and 50 µM Congo Red dye) was measured using a BMG FLUOstar OPTIMA plate reader. Values were normalized to the mean of the absorbances at 485 and 450 nm. Thioflavin T fluorescence of peptides/nucleic acids in 10 mM MES buffer was measured as previously described [23]. The insulin amyloid positive control was prepared by incubating 1 mM bovine pancreas insulin (Sigma, UK) in 10 mM hydrochloric acid for 3 days at 65°C followed by neutralization to pH 7 with NaOH. 50 µM insulin amyloid was used in assays.

### Time-resolved Thioflavin T assay

The measurements were taken on a BMG FLUOStar as described above (Nilsson 2002). The buffer was 150 mM NaCl in 10 mM MES pH 6.5 (+1 net charge on (HL)_3_, −3 net charge on (El)_3_), including 50 µM Thioflavin T. Of the DNA solution in buffer, 100 µl were injected at 420 µl per second to the 100 µl peptide solution in buffer, yielding 500 µM (HL)_3_+500 µM DNA final concentrations (1∶1 charge ratio). The temperature was recorded, but not controlled by the instrument, and was in the range of 27 to 30°C. The full program consists of the following steps:

The background level was determined by 5 measurements at 5 measurements per second followed by the injection of the salmon testes DNA within 1.7 seconds and a short orbital shaking for mixing (1 s). The ThT fluorescence immediately after injection and shaking was recorded in 200 ms steps for 40 s (200 data points), followed by 5 s steps for 225 s (45 data points).

### X-ray fibre diffraction

Drops of ANA complex solutions were placed between 2 beeswax-covered capillaries and allowed to dry to align the fibrillar material. Starting gels were formed from 5 mM (KL)_5_, 5 mM DNA, 10 mM HEPES pH 7, 150 mM NaCl; 16 mM (HL)_5_, 10 mM poly(A) RNA pH 6; 1.6 mM TVQFHMH, 1.6 mM DNA, 10 mM MES pH 5.0; 10 mM (KL)_3_, 10 mM DNA; 5 mM (HL)_3_, 5 mM DNA, 10 mM MES pH 6.2; 1.6 mM TVQFHMH (gel formed after titration to pH 11 then down to pH 6 using NaOH and HCl respectively); 20 mM unbuffered DNA; 20 mM unbuffered single-stranded DNA (DNA denatured by heating to 95°C and rapid cooling on ice water immediately before stalk preparation); 16 mM TVQFHMH, 10 mM poly(A) RNA pH 6.0; 16 mM (KL)_3_, 10 mM DNA (unbuffered). The resulting stalks were placed vertically in the x-ray beam of a Rigaku RU-H3R rotating anode x-ray generator (Cu K_α_ with 1.5418 Å wavelength), and the diffraction pattern was recorded on a Rigaku R-AXIS IV flat plate detector with 180 or 300 mm sample to detector distance and an exposure time of 10 to 20 minutes. The diffraction pattern was extracted with ImageJ [38] from the proprietary file format used by the Rigaku CrystalClear software (version 1.40) and the Bragg spacings determined with WCEN [39], [40].

### Transmission Electron Microscopy

The following ANA complexes were formed: 0.2 mM (KL)_5_, 0.25 mM (KL)_4_ or 0.33 mM (KL)_3_ solutions with 1 mM DNA or poly(A) RNA, 10 mM HEPES pH 7.0 as indicated; 1 mM (HL)_5_ or (HL)_3_ or TVQFHMH with 1 mM DNA or poly(A) RNA, 10 mM MES at the indicated pHs; 5 mM (HL)_3_ or 0.25 mM (KL)_4_ with 5 mM or 1 mM poly(A) RNA with 10 mM MES pH 6.2 or 10 mM HEPES pH 7.0; 5 mM (HL)_3_ or (HL)_5_ or TVQFHMH or 1 mM (KL)_5_ with 5 mM DNA with 150 mM NaCl in 10 mM MES/HEPES at the pHs indicated; 5 mM TVQFHMH with 150 mM NaCl in 10 mM MES pH 6.5. Following a 1∶2 dilution in buffer when necessary the samples were adsorbed onto Pioloform-coated copper 50 meshes or carbon-coated Formvar-coated copper 200/300 meshes and stained with 2% uranyl acetate as previously described [23], and examined on a Philips EM 208 or Philips CM12 electron microscope.

### Confocal Microscopy

FITC-(HL)_3_ was spiked into (HL)_3_ (1∶20 ratio) to generate a 12 mM (HL)_3_ working solution. Salmon testis DNA was labelled by nick-translation incorporating ATTO 550 nucleotides using a nick-translation kit (Jena Biosciences, Germany). Unincorporated nucleotides were removed by gel filtration. ATTO550-DNA was spiked into salmon testis DNA (1∶20 ratio) yielding a 5 mM (phosphate) working solution. Equal volumes of 5 mM DNA and 12 mM (HL)_3_ solutions were mixed directly on the slide for imaging. A coverslip was added and the samples sealed with clear nail varnish before imaging. Pictures were taken with a Leica DM6000B upright microscope with a ×63 oil objective using a galvanometer-driven high precision Z-stage. The bleed-through controls were prepared as described above, but the DNA or the (HL)_3_ component were not spiked with the fluorophore-labelled derivative.

### Circular Dichroism

20 mM STVIIE in 20 mM glycine pH 10.0 was diluted 1∶25 in 20 mM glycine pH 2.6 (carboxy group buffering) to yield 0.8 mM peptide with a measured pH of 2.6. Where indicated, the 33mer DNA oligonucleotide was premixed with the dilution buffer to yield a final concentration of 242 nM (8 µM with respect to phosphate). Samples were analysed in parallel in matching cuvettes (1 mm path length) using an Applied Photophysics Chirascan Circular Dichroism spectrometer from 190 to 260 nm (1 nm step size, integration over 2.5 seconds per step) every 30 minutes for 24 hours at 20°C. Background of buffer without or with DNA (3 averaged spectra) was subtracted, and the spectra were set to 0 mdeg ellipticity at 260 nm to remove baseline shift.

### TR-FRET

DNA hybridization oligonucleotides used for TR-FRET (Donor probe: CTGGGTGTAGCTGATCTAAGATCGCTAAC**T**TCA with TGCTGAAGAGCTGAAGTTAGCGATCTTAGATCAGCTACACCCAGTCAC for an 11 bp overlap or CACTGACCACTTACTGATGCTCGATCGAATCTCGCTC for a 0 bp overlap; Acceptor probe: C**T**GGTGAATGACTACGAGCTAGCTTAGAGCGAG with GCTCTTCAGCACTCGCTCTAAGCTAGCTCGTAGTCATTCACCAGTCAC for an 11 bp overlap or TGAAGTTAGCGATCTTAGATCAGCTACACCCAGTCAC for a 0 bp overlap) were synthesized with label on the underlined bases where ATTO 488 and ATTO 550 were present on donor and acceptor oligonucleotides respectively. Donor and acceptor oligonucleotide pairs were cartridge-purified (Biomers.net, Germany) and separately hybridized in 150 mM NaCl to generate 50 µM stock solutions by heating to 95°C followed by slow cooling.

ANA complexes in 10 mM MES, 150 mM NaCl were mixed with an equal volume of the probe oligonucleotides to generate final concentrations of 1 mM peptide, 1 mM DNA, 10 mM MES, 150 mM NaCl at pHs shown. Both DNA hybridization probes were used at the same concentration, ranging from 20 to 800 nM. The proportion of donor: acceptor pair hybridization was determined by time-resolved FRET using time-correlated single photon counting (TCSPC). A laser source provided ∼50 ps pulses with 20 MHz repetition rate at 473 nm (Becker & Hickl GmbH, Germany). Photon counting electronics (SPC-150, Becker & Hickl GmbH, Germany) were connected to a Hamamatsu H7422P-40 photomultiplier. The time resolution of the system was ∼250 ps. The fluorescence signal was collected over a typical integration time of 30 seconds (10 to 300 seconds).

FRET occurs when donor fluorophore ATTO 488 and acceptor fluorophore ATTO 550 are brought within their Förster radius (6.4 nm; Atto-Tec, Germany) by hybridization of the probes. This causes a faster fluorescence decay of the FRET donor and a delayed rise and delayed decay of acceptor fluorescence. The amount of bound donor, i.e. amount of hybridization, was extracted from the time course of the fluorescence donor by dividing with a reference time course derived from samples containing donor alone before using an exponential model (eq. 1) for fitting using OriginPro 8 (OriginLab, USA).
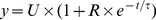
(1)This yielded the ratio *R* of bound to unbound donor probes, which was used to determine the amount of hybridization *f*, and the concentration of free donor *c_free_* from the starting concentration *c_0_*, via equations 2 and 3.
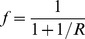
(2)

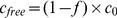
(3)The dissociation constant *K*_d_ was determined by fitting *f* over the concentration of free donor to a hyperbolic saturation curve (eq. 4) with *f*_max_ as maximum hybridization.
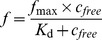
(4)To determine the NaCl-dependence of hybridization, the hybridization probes were used at 500 nM each, a concentration about 5× higher than the K_d_, thereby ensuring that only the concentration of NaCl was a limiting factor for hybridization. The NaCl concentration was varied from 1.125 mM to 1 M. Measurements were taken as described above. A Hill curve (equation 5) was fitted through the data points for visualization purposes.
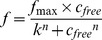
(5)


### Molecular Modelling

To visualize a possible structure of ANA complexes a molecular model of 16 (KL)_5_ peptide strands in cross-β configuration between 2 DNA molecules was created. The model was generated on a MacPro dual 2.66 GHz Xeon running Ubuntu 9.10. The peptides were built using the protein builder included in the Molecular Operating Environment (MOE 2008.10; Chemical Computing Group, Montreal, Quebec, Canada; http://www.chemcomp.com) as β-sheets. The individual peptides were manually oriented initially in an anti-parallel sheet conformation and then minimized using the Amber99 force field until a RMSD gradient of 0.05 kcal mol^−1^ Å^−1^ was reached, applying a distance constraint (2.5 Å to 3.5 Å – weight 100) to the appropriate hydrogen bonds between the individual peptides. Two sheets of 8 peptides were then positioned with the hydrophobic faces in contact and the system was further minimized as described above. Two double stranded DNA chains (PDB: 1BNA; [41]) were then put in contact with the polar faces of the peptide sheets and the system was minimized. To further relax the DNA-peptide complex, a short molecular dynamic simulation was carried out using Gromacs [42] with the Amber force field [43]. Initially, the system was solvated in a cubic box and appropriately neutralized. A steepest descent algorithm was used for the minimization of the solvated system and then a 250 ps molecular dynamics simulation was performed, in an NPT environment (293 K; 1 atm), applying a position restrain to all atoms. The coordinate of the final molecular dynamic step was analysed using VMD [44].

## Supporting Information

Figure S1 **Kinetics of amyloid formation of (HL)_3_ and (EL)_3_ ANA complexes.** A solution of peptide (red) or buffer (blue) was injected with salmon testes DNA to detect differences in the time course of Thioflavin T fluorescence. (**A**) The peptide (HL)_3_ in complex with salmon testes DNA shows a clear increase in ThT fluorescence after injection of salmon testes DNA within the 270 seconds of measurements. The sudden jumps and irregularities in ThT fluorescence levels may be explained by incomplete mixing or air bubbles created during the injection of the salmon testes DNA solution distorting the signal. (**B**) The peptide (EL)_3_ displays no increase in ThT fluorescence in the same time scale, showing that it is not able to form amyloid in complex with salmon testes DNA.
(TIFF)

Figure S2 **Additional TEM images.** Fibre formation of insulin amyloid and various ANA complexes as indicated. Scale bars are 300 nm.
(TIFF)

Figure S3 **Composite TEM image of a TVQFHMH-DNA fibre.** The sample was prepared from 5 mM TVQFHMH with 5 mM DNA at pH 5.0 and directly imaged without dilution. Scale bar is 1 µM×10 nm.
(TIFF)

Figure S4 **Model of a (KL)_5_-DNA complex as a guide to minimal fibre dimensions.** A model of a 16-peptide (KL)_5_ antiparallel amyloid fibril with two DNA strands was generated based on a previous model of an amyloid fibril formed from proteins with tandem 7mer hydrophobic - hydrophilic sequences [9]. Highly dynamic interactions between DNA and amyloid were observed during a short dynamic simulation. The DNA is 2.0 nm in diameter, the peptide backbone ∼3.1 nm (N- to C-terminal) and the β-sheet sandwich ∼2.6 nm across (ε-amino group to ε-amino group on lysine residues).
(TIFF)

Figure S5
**Hybridisation promotion by NaCl.** Hybridisation of the DNA probes reaches a saturation plateau at about 150 mM NaCl. 500 nM of donor and acceptor hybridisation probes were incubated with at 1.125 mM to 1 M NaCl at room temperature for 30 minutes before measurement. A Hill curve (red; eq. 5) with a Hill coefficient of *n* = 1.4±0.1 s.d. and a dissociation constant *k* = 53 mM±3 mM s.d. NaCl was fitted to the n = 9 data points (r^2^ = 0.996), indicating half-maximal saturation at 50 mM NaCl. There is some cooperativity as indicated by a Hill coefficient of *n*>1.
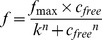
(5)
(TIFF)

Figure S6 **Peptide titration curves.** The peptides (HL)_3_ (**A**), (HL)_5_ (**B**), (KL)_3_ (**C**), (KL)_5_ (**D**) and TVQFHMH (**E**) were titrated with NaOH, and the peptide (EL)_3_ was titrated with HCl (**F**).
(TIFF)

Table S1 **Observations of gel formation for varying peptide : nucleic acid charge ratios.** Peptides (KL)_5_, TVQFHMH (TVQ) or (HL)_3_ were diluted to the indicated concentrations and mixed with equal volumes of salmon testes DNA at the indicated concentrations. All samples were prepared in 10 mM MES pH 6.8 giving the peptides the following net charges: (HL)_3_ and TVQFHMH +1, (KL)_5_ +5. Mixtures were monitored for strength and speed of gel formation. Seq, Sequence; Obs, Observations. Absence of gel is denoted by /.
(DOC)

Table S2 **pH dependence of gel formation by peptides.** Peptide net charges were altered by varying the pH of the buffer (10 mM MES, 150 mM NaCl). Peptides were mixed with equal volumes of salmon testes DNA or poly(A) RNA. Mixtures were monitored for strength and speed of gel formation. Decreasing NaCl concentration (150 mM to 75 mM) increased gel strength. Seq, Sequence; Obs, Observations. Absence of gel is denoted by /.
(DOC)

Table S3 **Gel formation by KL peptides and RNA from different sources.** Peptides (KL)_3_, (KL)_3.5_ or (KL)_4_ were diluted to the indicated concentrations and mixed with equal volumes of poly(A) RNA or Baker's Yeast (BY) RNA at the indicated concentrations. All samples were prepared in 10 mM MES pH 6.8. Mixtures were monitored for strength and speed of gel formation. Seq, Sequence; Obs, Observations. Absence of gel is denoted by /.
(DOC)

Table S4 **Reflexions of X-ray fibre diffraction experiments.** The Bragg spacings of the reflections are given in Å and are marked as meridional (m), equatorial (e), ring (r) or salt ring/spots (s). ds, double stranded; den., denatured. Reflections shown in italics: very weak. Unlabelled reflections, especially from the DNA samples, cannot be assigned to an orientation.
(DOC)

Table S5 **Diameters of ANA fibres.** Fibre diameters were determined using ImageJ from the fibres shown in figure 2 [38]. Three measurements were taken on clearly defined fibres with a distance of at least twice the diameter between measurement sites. Measurements were not taken of large diameter fibres/aggregates. Diameters are in nm ± standard deviation.
(DOC)
