# Supplementary figures and images for: Amyloid-Associated Nucleic Acid Hybridisation
Source: PLoS One. 2011 May 19;6(5):e19125. doi: 10.1371/journal.pone.0019125 (PMC3098241; doi:10.1371/journal.pone.0019125)

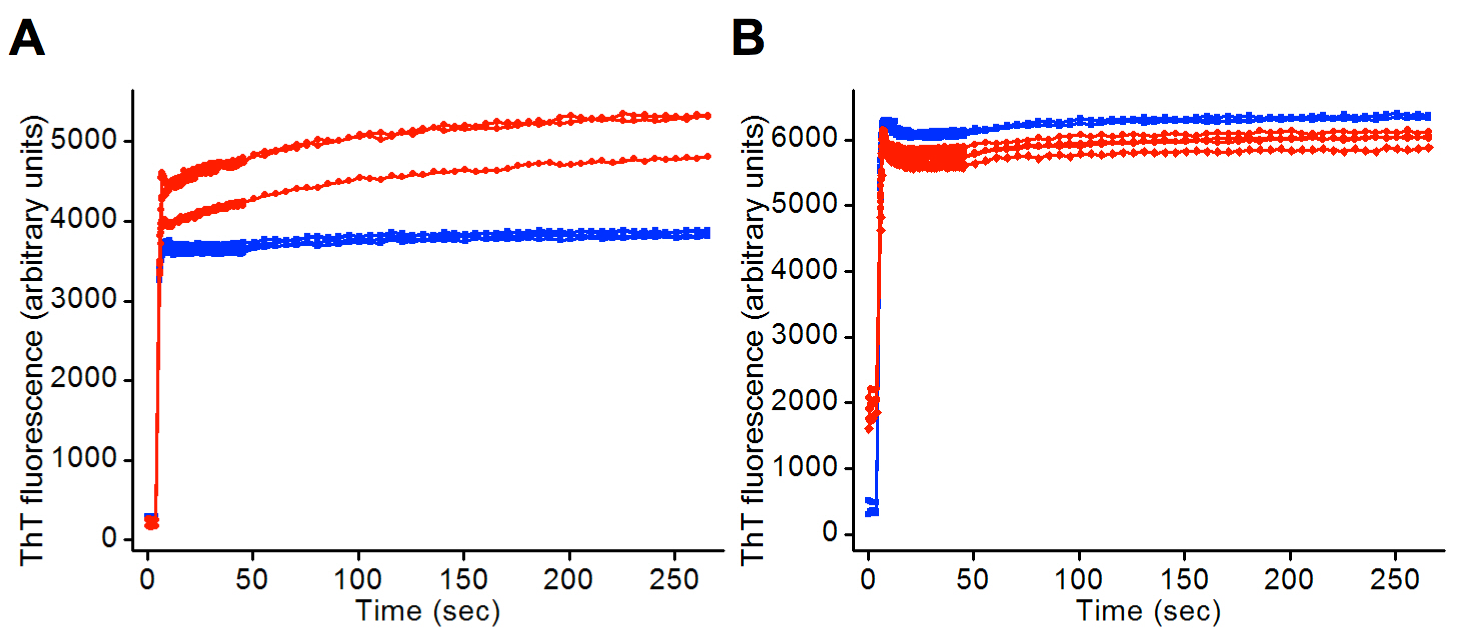

Supplement: Figure S1 — Kinetics of amyloid formation of (HL)3 and (EL)3 ANA complexes. A solution of peptide (red) or buffer (blue) was injected with salmon testes DNA to detect differences in the time course of Thioflavin T fluorescence. (A) The peptide (HL)3 in complex with salmon testes DNA shows a clear increase in ThT fluorescence after injection of salmon testes DNA within the 270 seconds of measurements. The sudden jumps and irregularities in ThT fluorescence levels may be explained by incomplete mixing or air bubbles created during the injection of the salmon testes DNA solution distorting the signal. (B) The peptide (EL)3 displays no increase in ThT fluorescence in the same time scale, showing that it is not able to form amyloid in complex with salmon testes DNA. (TIFF) [file pone.0019125.s001.tiff]

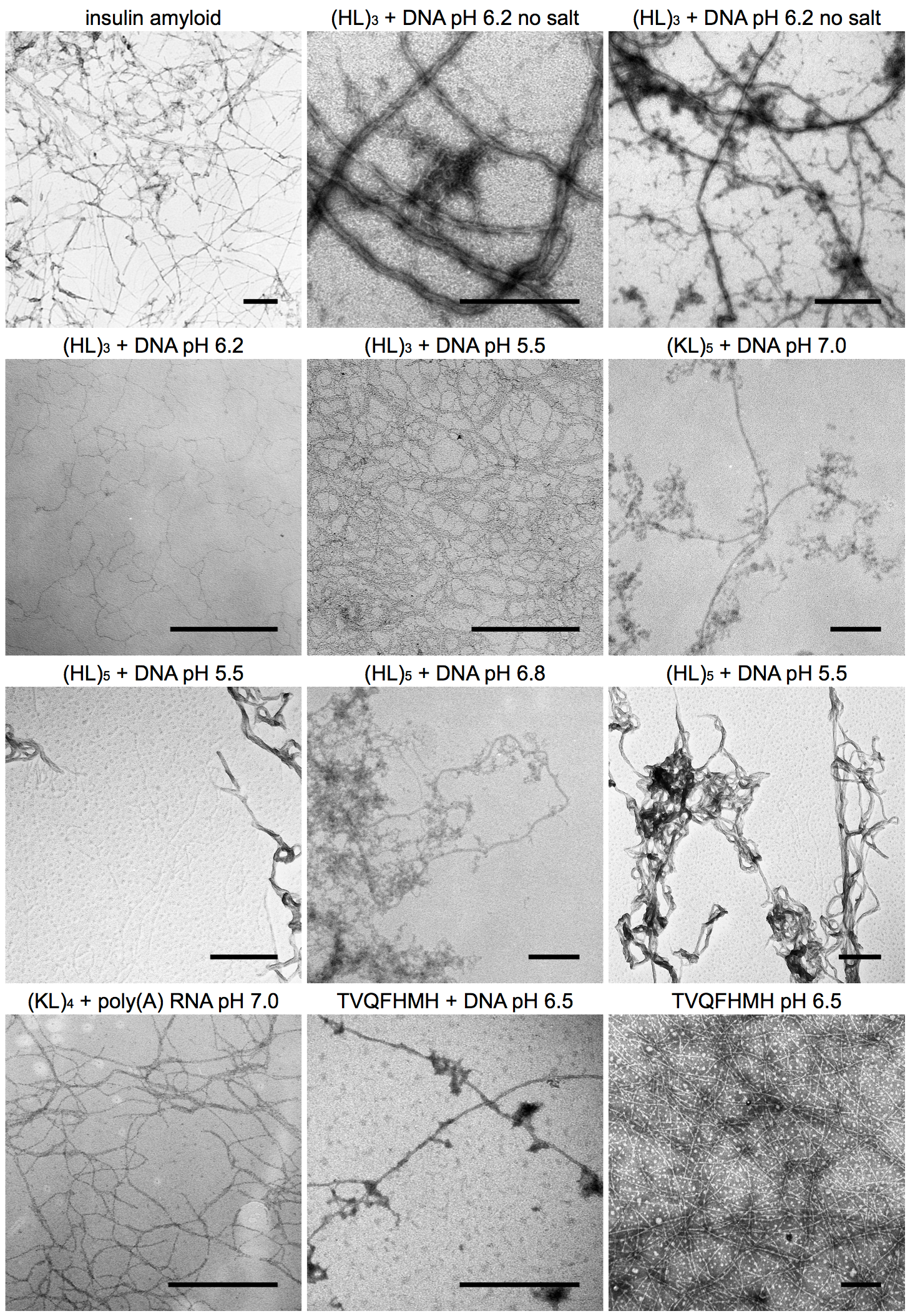

Supplement: Figure S2 — Additional TEM images. Fibre formation of insulin amyloid and various ANA complexes as indicated. Scale bars are 300 nm. (TIFF) [file pone.0019125.s002.tiff]

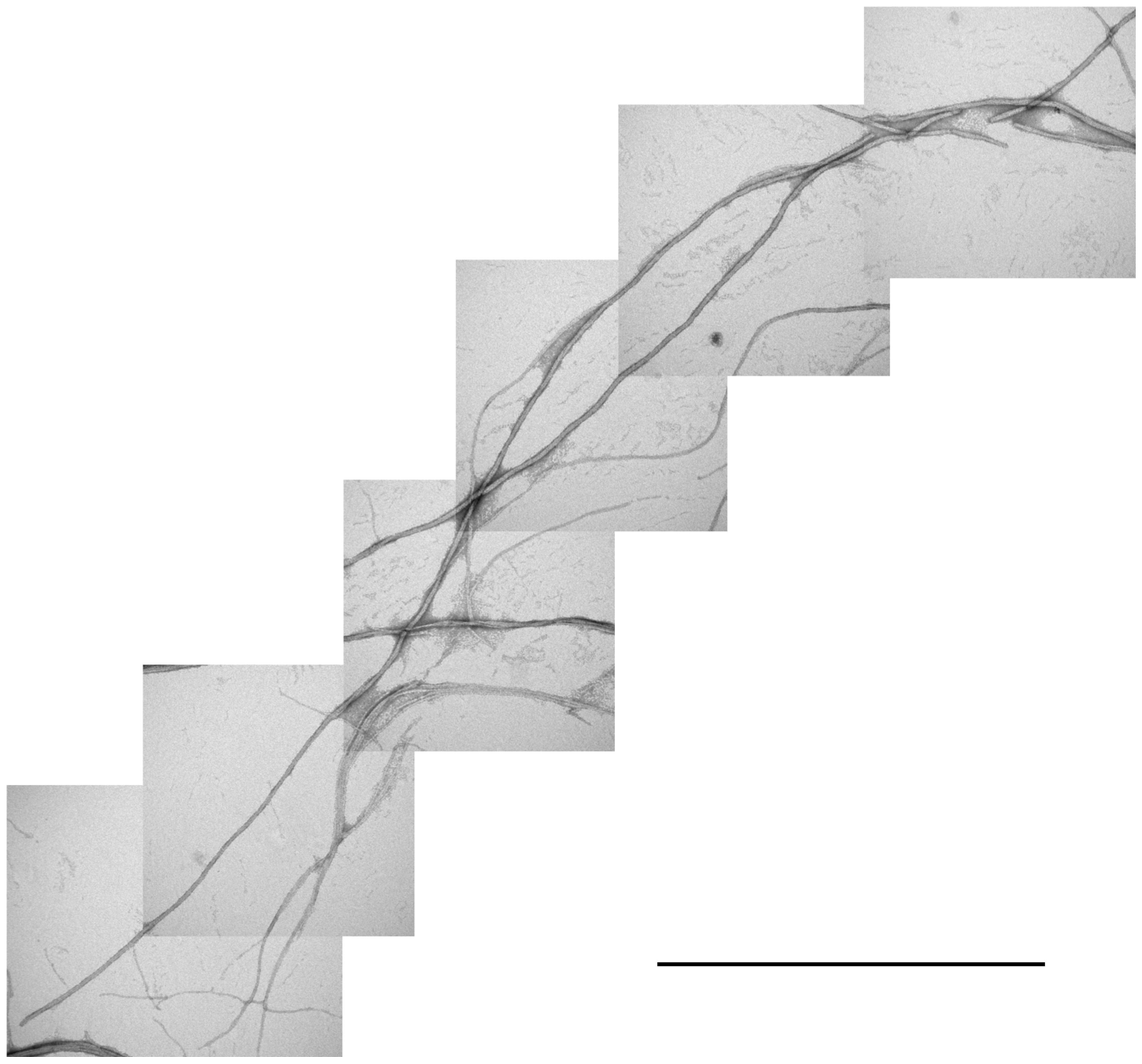

Supplement: Figure S3 — Composite TEM image of a TVQFHMH-DNA fibre. The sample was prepared from 5 mM TVQFHMH with 5 mM DNA at pH 5.0 and directly imaged without dilution. Scale bar is 1 µM×10 nm. (TIFF) [file pone.0019125.s003.tiff]

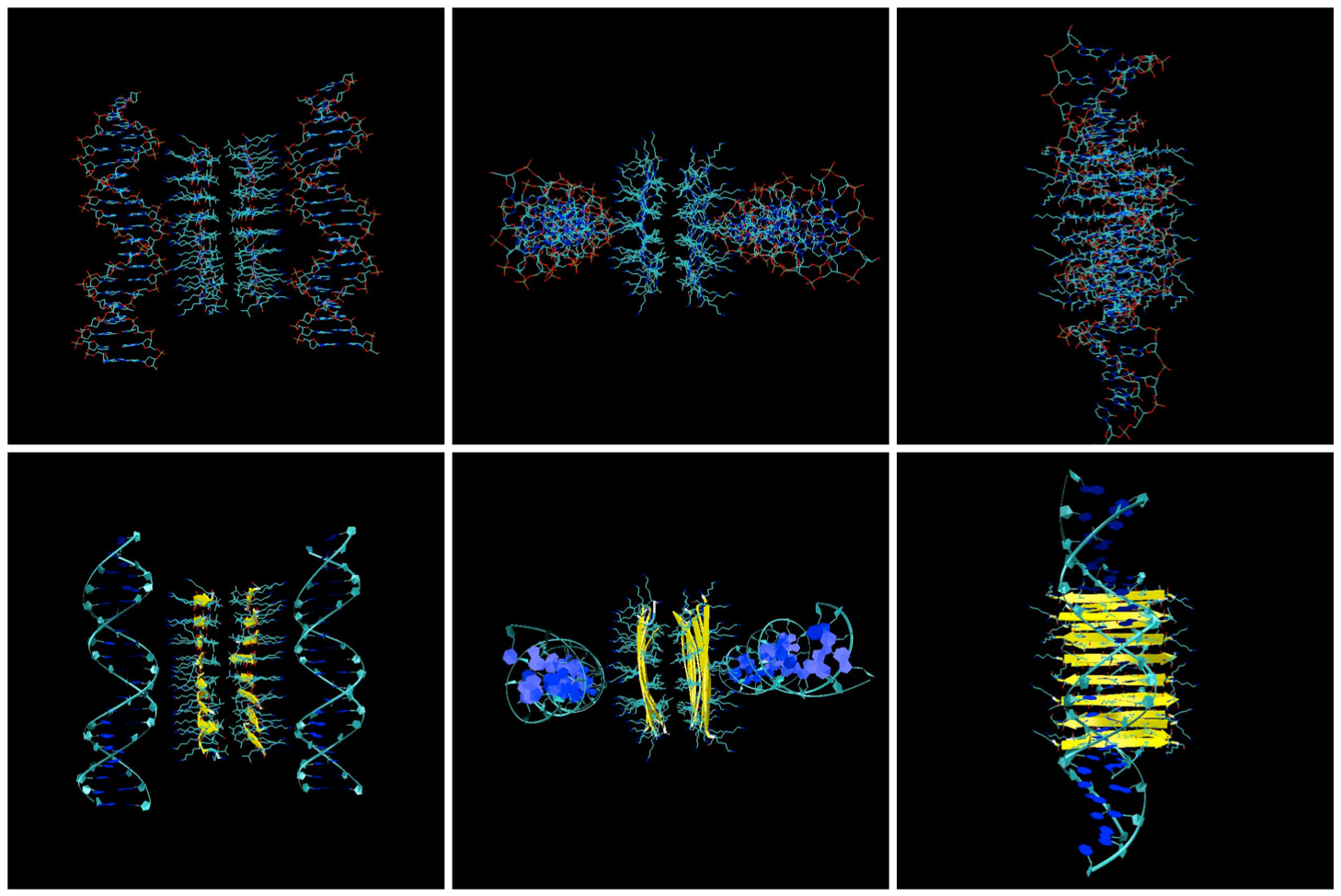

Supplement: Figure S4 — Model of a (KL)5-DNA complex as a guide to minimal fibre dimensions. A model of a 16-peptide (KL)5 antiparallel amyloid fibril with two DNA strands was generated based on a previous model of an amyloid fibril formed from proteins with tandem 7mer hydrophobic - hydrophilic sequences [9]. Highly dynamic interactions between DNA and amyloid were observed during a short dynamic simulation. The DNA is 2.0 nm in diameter, the peptide backbone ∼3.1 nm (N- to C-terminal) and the β-sheet sandwich ∼2.6 nm across (ε-amino group to ε-amino group on lysine residues). (TIFF) [file pone.0019125.s004.tiff]

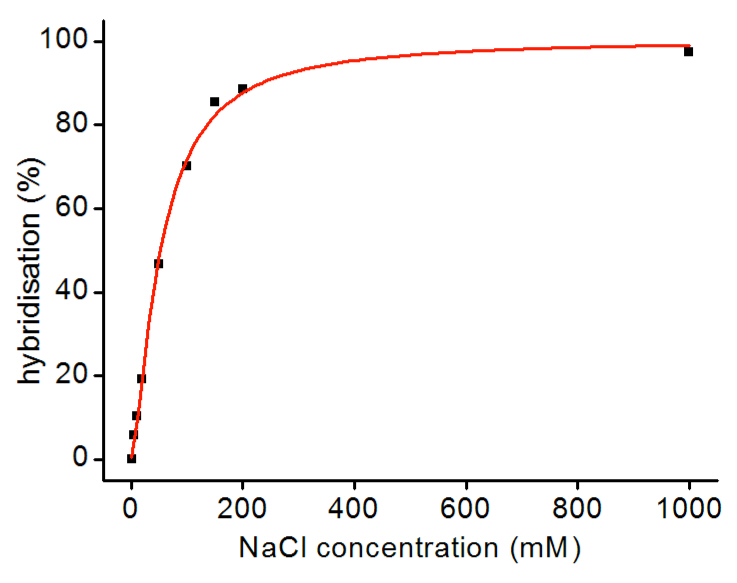

Supplement: Figure S5 — Hybridisation promotion by NaCl. Hybridisation of the DNA probes reaches a saturation plateau at about 150 mM NaCl. 500 nM of donor and acceptor hybridisation probes were incubated with at 1.125 mM to 1 M NaCl at room temperature for 30 minutes before measurement. A Hill curve (red; eq. 5) with a Hill coefficient of n = 1.4±0.1 s.d. and a dissociation constant k = 53 mM±3 mM s.d. NaCl was fitted to the n = 9 data points (r2 = 0.996), indicating half-maximal saturation at 50 mM NaCl. There is some cooperativity as indicated by a Hill coefficient of n>1.(5) (TIFF) [file pone.0019125.s005.tiff]

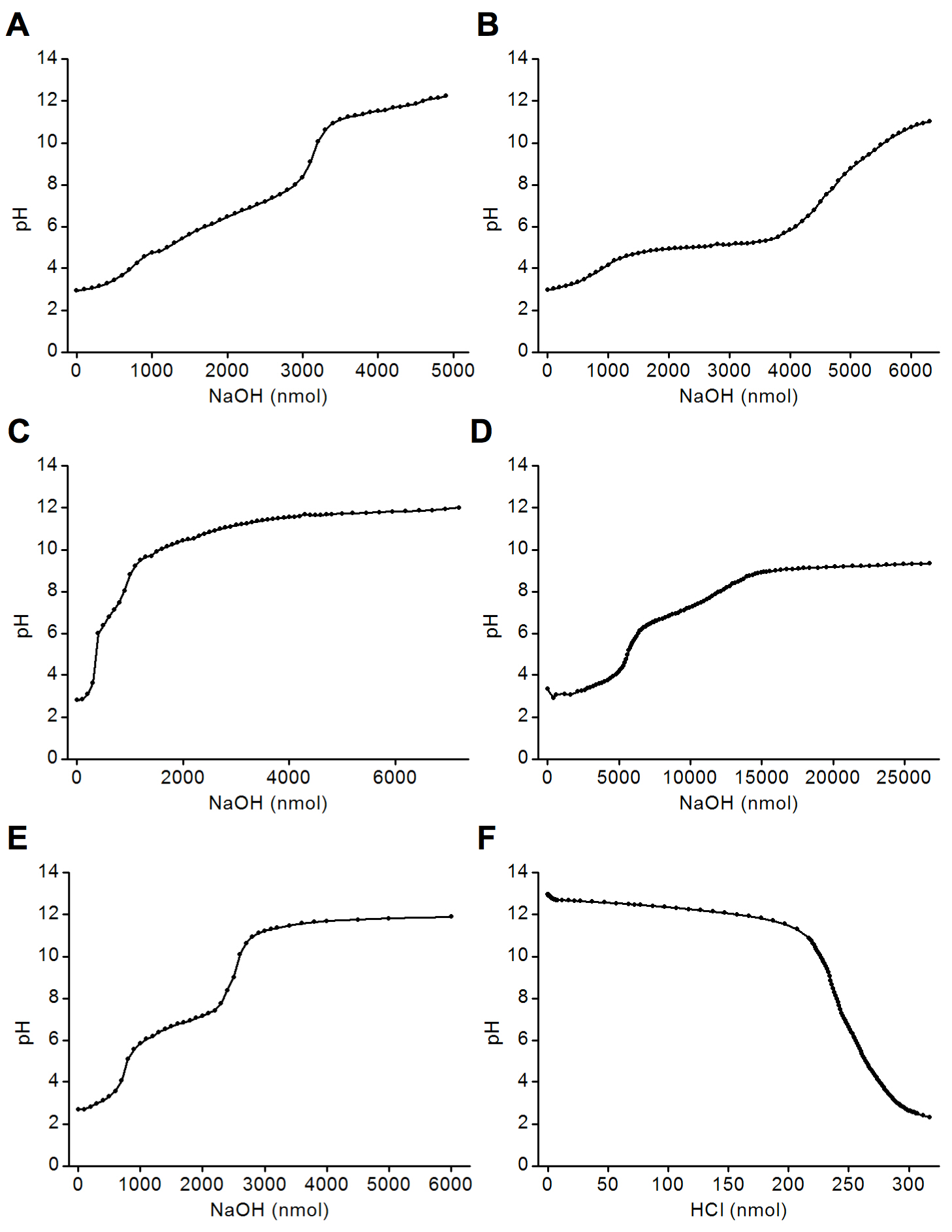

Supplement: Figure S6 — Peptide titration curves. The peptides (HL)3 (A), (HL)5 (B), (KL)3 (C), (KL)5 (D) and TVQFHMH (E) were titrated with NaOH, and the peptide (EL)3 was titrated with HCl (F). (TIFF) [file pone.0019125.s006.tiff]
