# Supplementary material for: Amyloid-Associated Nucleic Acid Hybridisation
Source: PLoS One. 2011 May 19;6(5):e19125. doi: 10.1371/journal.pone.0019125 (PMC3098241; doi:10.1371/journal.pone.0019125)
Supplement: Table S1 — Observations of gel formation for varying peptide : nucleic acid charge ratios. Peptides (KL)5, TVQFHMH (TVQ) or (HL)3 were diluted to the indicated concentrations and mixed with equal volumes of salmon testes DNA at the indicated concentrations. All samples were prepared in 10 mM MES pH 6.8 giving the peptides the following net charges: (HL)3 and TVQFHMH +1, (KL)5 +5. Mixtures were monitored for strength and speed of gel formation. Seq, Sequence; Obs, Observations. Absence of gel is denoted by /. (DOC) [file pone.0019125.s007.doc]

**Table S1. Observations of gel formation for varying peptide : nucleic acid charge ratios.**

| **Peptide** | | | **Phosphate** | |  |  |  |
| --- | --- | --- | --- | --- | --- | --- | --- |
| **Seq** | **Final Conc. Peptide (mM)** | **Final Conc. Peptide wrt Charge (mM)** | **Source** | **Final Conc. wrt P (mM)** | **Peptide:P** | **NaCl (mM)** | **Obs** |
| (KL)_5_ | 0 | 0 | ST DNA | 10 | 0:10 | 150 | / |
| " | 1 | 5 | ST DNA | 9 | 1:9 | " | / |
| " | 2 | 10 | ST DNA | 8 | 1:4 | " | weak gel |
| " | 3 | 15 | ST DNA | 7 | 3:7 | " | gel |
| " | 4 | 20 | ST DNA | 6 | 2:3 | " | gel |
| " | 5 | 25 | ST DNA | 5 | 1:1 | " | gel |
| " | 6 | 30 | ST DNA | 4 | 3:2 | " | strong gel |
| " | 7 | 35 | ST DNA | 3 | 7:3 | " | strong gel |
| " | 8 | 40 | ST DNA | 2 | 4:1 | " | strong gel |
| " | 9 | 45 | ST DNA | 1 | 9:1 | " | strong gel |
| " | 10 | 50 | ST DNA | 10 | 1:1 | " | strong gel |
| " | 10 | 50 | - | 0 | 10:0 | " | / |
| " | 1 | 5 | NaPO_4_ | 5 | 1:1 | " | / |
| " | 9 | 45 | NaPO_4_ | 100 | 1:2 | " | / |
| " | 5 | 25 | poly(A)RNA | 5 | 5:1 | " | weak gel |
| TVQ | 0 | 0 | ST DNA | 10 | 0:10 | " | / |
| " | 1 | 1 | ST DNA | 9 | 1:9 | " | / |
| " | 2 | 2 | ST DNA | 8 | 1:4 | " | gel |
| " | 3 | 3 | ST DNA | 7 | 3:7 | " | gel |
| " | 4 | 4 | ST DNA | 6 | 2:3 | " | gel |
| " | 5 | 5 | ST DNA | 5 | 1:1 | " | gel |
|  | 6 | 6 | ST DNA | 4 | 3:2 | " | strong gel |
| " | 7 | 7 | ST DNA | 3 | 7:3 | " | strong gel |
| " | 8 | 8 | ST DNA | 2 | 4:1 | " | gel |
| " | 9 | 9 | ST DNA | 1 | 9:1 | " | gel |
| “ | 10 | 10 | ST DNA | 10 | 1:1 | " | strong gel |
| " | 10 | 10 |  | 0 | 10:0 | " | / |
| " | 5 | 5 | NaPO_4_ | 5 | 1:1 | " | / |
| " | 0 | 0 | poly(A) RNA | 10 | 0:10 | " | / |
| " | 1 | 1 | poly(A) RNA | 9 | 1:9 | " | / |
| " | 2 | 2 | poly(A) RNA | 8 | 1:4 | " | / |
| " | 3 | 3 | poly(A) RNA | 7 | 3:7 | " | / |
| " | 4 | 4 | poly(A) RNA | 6 | 2:3 | " | weak gel, slow to form |
| " | 5 | 5 | poly(A) RNA | 5 | 1:1 | " | weak gel, slow to form |
|  | 6 | 6 | poly(A) RNA | 4 | 3:2 | " | weak gel, slow to form |
| " | 7 | 7 | poly(A) RNA | 3 | 7:3 | " | weak gel, slow to form |
| " | 8 | 8 | poly(A) RNA | 2 | 4:1 | " | weak gel, slow to form |
| " | 9 | 9 | poly(A) RNA | 1 | 9:1 | " | weak gel, slow to form |
| " | 10 | 10 | poly(A) RNA | 10 | 1:1 | " | weak gel, slow to form |
| " | 10 | 10 | - | 0 | 10:0 | " | / |
| (HL)_3_ | 0 | 0 | ST DNA | 10 | 0:10 | 150 | / |
| " | 1 | 1 | ST DNA | 9 | 1:9 | " | / |
| " | 2 | 2 | ST DNA | 8 | 1:4 | " | / |
| " | 3 | 3 | ST DNA | 7 | 3:7 | " | / |
| " | 4 | 4 | ST DNA | 6 | 2:3 | " | / |
| " | 5 | 5 | ST DNA | 5 | 1:1 | " | / |
| " | 6 | 6 | ST DNA | 4 | 3:2 | " | Very Weak Gel |
| " | 7 | 7 | ST DNA | 3 | 7:3 | " | Very Weak Gel |
| " | 8 | 8 | ST DNA | 2 | 4:1 | " | Very Weak Gel |
| " | 9 | 9 | ST DNA | 1 | 9:1 | " | Very Weak Gel |
| " | 10 | 10 | ST DNA | 10 | 1:1 | " | weak gel |
| " | 10 | 10 | - | 0 | 10:0 | " | / |
| " | 5 | 5 | NaPO_4_ | 5 | 1:1 | " | / |
| " | 5 | 5 | poly(A) RNA | 5 | 1:1 | " | / |

Peptides (KL)_5_, TVQFHMH (TVQ) or (HL)_3_ were diluted to the indicated concentrations and mixed with equal volumes of salmon testes DNA at the indicated concentrations. All samples were prepared in 10mM MES pH 6.8 giving the peptides the following net charges: (HL)_3_ and TVQFHMH +1, (KL)_5_ +5. Mixtures were monitored for strength and speed of gel formation. Seq, Sequence; Obs, Observations. Absence of gel is denoted by /.
