# Supplementary material for: Amyloid-Associated Nucleic Acid Hybridisation
Source: PLoS One. 2011 May 19;6(5):e19125. doi: 10.1371/journal.pone.0019125 (PMC3098241; doi:10.1371/journal.pone.0019125)
Supplement: Table S2 — pH dependence of gel formation by peptides. Peptide net charges were altered by varying the pH of the buffer (10 mM MES, 150 mM NaCl). Peptides were mixed with equal volumes of salmon testes DNA or poly(A) RNA. Mixtures were monitored for strength and speed of gel formation. Decreasing NaCl concentration (150 mM to 75 mM) increased gel strength. Seq, Sequence; Obs, Observations. Absence of gel is denoted by /. (DOC) [file pone.0019125.s008.doc]

**Table S2. pH dependence of gel formation by peptides**.

| **Peptide** | | | | | **Phosphate** | | |  |  |  |
| --- | --- | --- | --- | --- | --- | --- | --- | --- | --- | --- |
| **Seq** | **Final Conc. Peptide (mM)** | **Final Conc. Peptide wrt Charge (mM)** | **Charge** | **pH** | **Source** | **Final Conc. wrt P mM** | **pH** | **Final NaCl (mM)** | **Peptide Charge:P** | **Obs** |
| (HL)_3_ | 12 | 36 | 3+ | 5 | ST DNA | 5 | 5 | 75 | 7.2:1 | / |
| " | 12 | 24 | 2+ | 6.2 | ST DNA | 5 | 6.2 | " | 4.8:1 | Strong Gel |
| " | 12 | 12 | 1+ | 6.5 | ST DNA | 5 | 6.5 | " | 2.4:1 | Strong Gel |
| " | 12 | 36 | 3+ | 5 | NaPO_4_ | 100 | 5 | " | 1:2.7 | / |
| " | 12 | 24 | 2+ | 6.2 | NaPO_4_ | 100 | 6.2 | " | 1:4.2 | / |
| " | 12 | 12 | 1+ | 6.5 | NaPO_4_ | 100 | 6.5 | " | 1:8.3 | / |
| " | 2.4 | 7.2 | 3+ | 5 | ST DNA | 5 | 5 | " | 1.4:1 | / |
| " | 2.4 | 4.8 | 2+ | 6.2 | ST DNA | 5 | 6.2 | " | 1:1 | Very Weak Gel |
| " | 2.4 | 2.4 | 1+ | 6.5 | ST DNA | 5 | 6.5 | " | 1:2 | Very Weak Gel |
| " | 5 | 15 | 3+ | 5 | ST DNA | 5 | 5 | 150 | 3:1 | / |
| " | 5 | 10 | 2+ | 6.2 | ST DNA | 5 | 6.2 | " | 2:1 | Weak Gel |
| “ | 5 | 5 | 1+ | 6.8 | ST DNA | 5 | 6.8 | " | 1:1 | Weak Gel |
| " | 5 | 15 | 3+ | 5 | NaPO_4_ | 100 | 5 | " | 1:6.7 | / |
| " | 5 | 10 | 2+ | 6.2 | NaPO_4_ | 100 | 6.2 | " | 1:10 | / |
| " | 5 | 5 | 1+ | 6.8 | NaPO_4_ | 100 | 6.8 | " | 1:20 | / |
| TVQ | 28.3 | 56.4 | 2+ | 5 | ST DNA | 5 | 5 | 75 | 11:1 | Gel |
| " | 28.3 | 28.3 | 1+ to 2+ | 6.2 | ST DNA | 5 | 6.2 | " | 5.6:1 | Strong Gel |
| " | 28.3 | 28.3 | 1+ | 6.5 | ST DNA | 5 | 6.5 | " | 5.6:1 | Very Strong |
| " | 28.3 | 56.4 | 2+ | 5 | NaPO_4_ | 100 |  | " | 1:1.8 | / |
| " | 28.3 | 28.3 | 1+ | 6.5 | NaPO_4_ | 100 |  | " | 1:3.5 | / |
| " | 5 | 10 | 2+ | 5 | ST DNA | 5 | 5 | 150 | 2:1 | Gel |
| " | 5 | 5 | 1+ to 2+ | 6.2 | ST DNA | 5 | 6.2 | " | 1:1 | Gel |
| (KL)_5_ | 5 | 25 | 5+ | 6.2 | ST DNA | 5 | 6.2 | " | 5:1 | Gel |
| " | 5 | 25 | 5+ | 6.2 | Poly(A) RNA | 5 | 6.2 | " | 5:1 | very weak gel |
| " | 5 | 25 | 5+ | 6.2 | NaPO_4_ | 100 | 6.2 | " | 1:4 | / |
| (HL)_3_ | 5 | 10 | 2+ | 6.2 | STDNA | 5 | 6.2 | 75 | 2:1 | Gel |
| “ | 5 | 10 | 2+ | 6.2 | STDNA | 5 | 6.2 | 150 | 2:1 | Weak gel |

Peptide net charges were altered by varying the pH of the buffer (10mM MES, 150mM NaCl). Peptides were mixed with equal volumes of salmon testes DNA or poly(A) RNA. Mixtures were monitored for strength and speed of gel formation. Decreasing NaCl concentration (150mM to 75mM) increased gel strength. Seq, Sequence; Obs, Observations. Absence of gel is denoted by /.
