# Supplementary material for: Amyloid-Associated Nucleic Acid Hybridisation
Source: PLoS One. 2011 May 19;6(5):e19125. doi: 10.1371/journal.pone.0019125 (PMC3098241; doi:10.1371/journal.pone.0019125)
Supplement: Table S3 — Gel formation by KL peptides and RNA from different sources. Peptides (KL)3, (KL)3.5 or (KL)4 were diluted to the indicated concentrations and mixed with equal volumes of poly(A) RNA or Baker's Yeast (BY) RNA at the indicated concentrations. All samples were prepared in 10 mM MES pH 6.8. Mixtures were monitored for strength and speed of gel formation. Seq, Sequence; Obs, Observations. Absence of gel is denoted by /. (DOC) [file pone.0019125.s009.doc]

**Table S3. Gel formation by KL peptides and RNA from different sources**.

| **Peptide** | | | | | | | | | **Phosphate** | | |  |  |  |
| --- | --- | --- | --- | --- | --- | --- | --- | --- | --- | --- | --- | --- | --- | --- |
| **Seq** | **Final Conc. Peptide (mM)** | | **Final Conc. Peptide wrt Charge (mM)** | | **Charge** | | **pH** | | **Source** | **Final Conc. (wrt P) mM** | **pH** | **Final NaCl (mM)** | **Peptide Charge:P** | **Obs** |
| (KL)_3_ | | 1.4 | | 4.2 | | 3+ | | 6.8 | Poly(A) RNA | 7 | 6.8 | 0 | 1:1.6 | gel |
|  | |  | | 4.2 | | 3+ | | 6.8 | BY RNA | 7 | 6.8 | 0 | 1:1.6 | / |
| (KL)_3.5_ | | 1.2 | | 4.8 | | 4+ | | 6.8 | Poly(A) RNA | 7 | 6.8 | 0 | 1:1.5 | gel |
|  | |  | | 4.8 | | 4+ | | 6.8 | BY RNA | 7 | 6.8 | 0 | 1:1.5 | / |
| (KL)_4_ | | 1 | | 4 | | 4+ | | 6.8 | Poly(A) RNA | 7 | 6.8 | 0 | 1:1.7 | gel |
|  | |  | | 4 | | 4+ | | 6.8 | BY RNA | 7 | 6.8 | 0 | 1:1.7 | gel |

Peptides (KL)_3_, (KL)_3.5_ or (KL)_4_ were diluted to the indicated concentrations and mixed with equal volumes of poly(A) RNA or Baker’s Yeast (BY) RNA at the indicated concentrations. All samples were prepared in 10mM MES pH 6.8. Mixtures were monitored for strength and speed of gel formation. Seq, Sequence; Obs, Observations. Absence of gel is denoted by /.
