# Supplementary material for: Amyloid-Associated Nucleic Acid Hybridisation
Source: PLoS One. 2011 May 19;6(5):e19125. doi: 10.1371/journal.pone.0019125 (PMC3098241; doi:10.1371/journal.pone.0019125)
Supplement: Table S4 — Reflexions of X-ray fibre diffraction experiments. The Bragg spacings of the reflections are given in Å and are marked as meridional (m), equatorial (e), ring (r) or salt ring/spots (s). ds, double stranded; den., denatured. Reflections shown in italics: very weak. Unlabelled reflections, especially from the DNA samples, cannot be assigned to an orientation. (DOC) [file pone.0019125.s010.doc]

**Table S4. Reflexions of X-ray fibre diffraction experiments.**

| **TVQ** | | | | | | **(HL)_3_** | | | | **(KL)_5_** | | **(KL)_3_** | | **ds DNA** | | **den. DNA** | |
| --- | --- | --- | --- | --- | --- | --- | --- | --- | --- | --- | --- | --- | --- | --- | --- | --- | --- |
| **alone** | | **+ DNA** | | **+ RNA** | | **+ RNA** | | **+ DNA** | | **+ DNA** | | **+ DNA** | |  | |  | |
| 16.4 | e | 16.1 | e | 32.6 | e | 50.0 | e | 23.8 | e | 13.9 | e | 20.9 | e | 16.3 |  | 3.4 | m |
| 14.5 | e | 10.6 | e | 18.0 | e | 24.8 | e | 11.7 | e | 9.4 | e | 14.2 | e | 14.8 |  | 5.5 | r |
| 10.5 | e | 7.5 | e | 11.5 | e | 12.5 | e | 12.5 | e | 5.6 | e | 11.4 | m | 11.8 |  | 4.3 |  |
| 7.7 | e | 6.5 | e | 10.3 | e | 9.5 | e | 9.3 | m | 4.8 | m | 9.3 | e | 11.3 |  | 17.9 | e |
| 4.8 | m | 5.7 | e | 7.4 | e | 8.3 | m | 4.7 | m | 4.6 | m | 7.6 | e | 10.5 |  | 9.0 |  |
| 4.6 | m | 5.5 | m | 5.7 | e | 6.2 | e | 4.5 | m | 3.9 | m | 6.8 | e | 17.9 | e |  |  |
| 4.2 | m | 5.0 | e | 4.9 | m | 4.8 | m | 4.2 | m | 3.3 | s | 5.6 | m,e | 11.4 | e |  |  |
| 3.7 | m | 4.8 | m | 4.7 | m | 4.5 | m | 3.9 | r | 2.8 | s | 4.6 | m | 10.9 | e |  |  |
| 3.3 | s | 4.6 | m | 4.5 | e | 4.2 | e | 3.3 | s |  |  | 3.8 | m | 10.6 |  |  |  |
| 3.1 | r | 4.3 | e | 4.2 | m | 3.3 | s | 2.8 | s |  |  |  |  | 9.5 |  |  |  |
| 2.8 | s | 4.1 | m | 3.7 | m | 2.9 | s | 2.0 | s |  |  |  |  | 8.1 |  |  |  |
|  |  | 3.9 | m |  |  |  |  |  |  |  |  |  |  | 7.4 |  |  |  |
|  |  | 3.71 | m |  |  |  |  |  |  |  |  |  |  | 7.3 | e |  |  |
|  |  | 3.65 | m |  |  |  |  |  |  |  |  |  |  | 6.5 |  |  |  |
|  |  | 3.5 | e |  |  |  |  |  |  |  |  |  |  | 5.5 | e |  |  |
|  |  | 3.5 | e |  |  |  |  |  |  |  |  |  |  | 4.3 | e |  |  |
|  |  | 3.3 | s |  |  |  |  |  |  |  |  |  |  | 4.1 | e |  |  |
|  |  | 2.8 | s |  |  |  |  |  |  |  |  |  |  | 4.5 | e |  |  |
|  |  | 3.0 | e |  |  |  |  |  |  |  |  |  |  | 3.0 |  |  |  |
|  |  | *2.7* | *m* |  |  |  |  |  |  |  |  |  |  | 5.0 |  |  |  |
|  |  | *2.6* | *m* |  |  |  |  |  |  |  |  |  |  | 4.8 |  |  |  |
|  |  | *2.52* | *m* |  |  |  |  |  |  |  |  |  |  | 4.6 |  |  |  |
|  |  | *2.48* | *m* |  |  |  |  |  |  |  |  |  |  | 4.5 |  |  |  |
|  |  | *2.41* | *m* |  |  |  |  |  |  |  |  |  |  | 4.3 |  |  |  |
|  |  | *2.37* | *m* |  |  |  |  |  |  |  |  |  |  | 4.2 |  |  |  |
|  |  |  |  |  |  |  |  |  |  |  |  |  |  | 4.1 |  |  |  |
|  |  |  |  |  |  |  |  |  |  |  |  |  |  | 3.8 |  |  |  |
|  |  |  |  |  |  |  |  |  |  |  |  |  |  | 3.6 |  |  |  |
|  |  |  |  |  |  |  |  |  |  |  |  |  |  | 3.5 |  |  |  |
|  |  |  |  |  |  |  |  |  |  |  |  |  |  | 3.2 |  |  |  |
|  |  |  |  |  |  |  |  |  |  |  |  |  |  | 3.0 | m | |  |
|  |  |  |  |  |  |  |  |  |  |  |  |  |  | 3.2 |  |  |  |
|  |  |  |  |  |  |  |  |  |  |  |  |  |  | 3.8 |  |  |  |

The Bragg spacings of the reflections are given in Å and are marked as meridional (m), equatorial (e), ring (r) or salt ring/spots (s). ds, double stranded; den., denatured. Reflections shown in italics: very weak. Unlabelled reflections, especially from the DNA samples, cannot be assigned to an orientation.
