# Supplementary material for: Amyloid-Associated Nucleic Acid Hybridisation
Source: PLoS One. 2011 May 19;6(5):e19125. doi: 10.1371/journal.pone.0019125 (PMC3098241; doi:10.1371/journal.pone.0019125)
Supplement: Table S5 — Diameters of ANA fibres. Fibre diameters were determined using ImageJ from the fibres shown in figure 2 [38]. Three measurements were taken on clearly defined fibres with a distance of at least twice the diameter between measurement sites. Measurements were not taken of large diameter fibres/aggregates. Diameters are in nm ± standard deviation. (DOC) [file pone.0019125.s011.doc]

**Table S5. Diameters of ANA fibres.**

| **(KL)_5_ + DNA**  **pH 6.8** | **(KL)_4_ + RNA** | **(KL)_3_ + RNA** | **(HL)_5_ + DNA**  **pH 5.5** | **(HL)_3_ + DNA**  **pH 6.2** | **TVQ + DNA**  **pH 6.5** | **TVQ**  **pH 6.5** | **insulin amyloid** |
| --- | --- | --- | --- | --- | --- | --- | --- |
| 8.1 ± 1.1 | 5.9 ± 1.2 | 4.4 ± 0.4 | 5.8 ± 0.3 | 3.0 ± 0.3 | 9.9 ± 1.0 | 6.4 ± 0.5 | 7.5 ± 0.9 |
| 11.6 ± 0.9 | 7.0 ± 0.7 | 5.4 ± 0.4 | 6.3 ± 0.3 | 3.3 ± 0.3 | 15.4 ± 1.8 | 8.2 ± 1.0 | 7.6 ± 0.9 |
| 11.7 ± 0.7 | 7.1 ± 0.4 | 5.4 ± 0.4 | 6.7 ± 0.3 | 3.3 ± 0.6 | 18.2 ± 1.1 | 8.4 ± 0.9 | 9.1 ± 0.4 |
| 15.0 ± 3.0 | 7.6 ± 0.6 | 5.5 ± 0.5 | 10.6 ± 0.3 | 3.6 ± 0.1 | 19.5 ± 1.6 | 8.8 ± 0.3 | 9.4 ± 0.9 |
| 16.1 ± 2.6 | 8.3 ± 0.9 | 5.7 ± 0.8 | 13.2 ± 1.0 | 4.0 ± 0.8 | 19.8 ± 2.6 | 9.2 ± 1.7 | 10.1 ± 1.5 |
| 18.5 ± 3.4 | 9.0 ± 2.2 | 5.9 ± 0.9 | 18.2 ± 1.3 | 4.1 ± 0.3 | 20.2 ± 1.5 | 9.7 ± 1.5 | 11.1 ± 1.6 |

Fibre diameters were determined using ImageJ from the fibres shown in figure 2 *(*33). Three measurements were taken on clearly defined fibres with a distance of at least twice the diameter between measurement sites. Measurements were not taken of large diameter fibres/aggregates. Diameters are in nm ± standard deviation.
